# Conservative treatment in patients with an acute lumbosacral radicular syndrome: design of a randomised clinical trial [ISRCTN68857256]

**DOI:** 10.1186/1471-2474-5-39

**Published:** 2004-11-09

**Authors:** Pim AJ Luijsterburg, Arianne P Verhagen, Raymond WJG Ostelo, Hans JMM van den Hoogen, Wilco C Peul, Cees JJ Avezaat, Bart W Koes

**Affiliations:** 1 General Practice, University Medical Center Rotterdam (Erasmus MC), PO Box 1736, 3000 DR Rotterdam, The Netherlands; 2 EMGO Institute, University Medical Center (VU), Van der boechorsstraat 7, 1081 BT Amsterdam, The Netherlands; 3 General Practice, J v/d Diesduncstraat 18, 5721 VM Asten, The Netherlands; 4 Neurosurgery, Leids University Medical Center (LUMC), PO Box 9600, 2300 RC Leiden, The Netherlands; 5 Neurosurgery, University Medical Center Rotterdam (Erasmus MC), PO Box 1736, 3000 DR Rotterdam, The Netherlands

## Abstract

**Background:**

The objective is to present the design of randomised clinical trial (RCT) on the effectiveness of physical therapy added to general practitioners management compared to general practitioners management only in patients with an acute lumbosacral radicular syndrome (also called sciatica).

**Methods/Design:**

Patients in general practice diagnosed with an acute (less than 6 weeks) lumbosacral radicular syndrome and an age above 18 years are eligible for participation. The general practitioners treatment follows their clinical guideline. The physical therapy treatment will consist of patient education and exercise therapy. The primary outcome measure is patients reported global perceived effect. Secondary outcome measures are severity of complaints, functional status, health status, fear of movement, medical consumption, sickness absence, costs and treatment preference. The follow-up is 52 weeks.

**Discussion:**

Treatment by general practitioners and physical therapists in this study will be transparent and not a complete "black box". The results of this trial will contribute to the decision of the general practitioner regarding referral to physical therapy in patients with an acute lumbosacral radicular syndrome.

## Background

### Why a design article?

Publishing the design of the trial has several advantages. It may prevent publication bias [1]. A study producing positive results seems more likely to be published than a study that reports no or negative results [2,3]. Also, the study can be included in systematic reviews because data can be retrieved from the researcher [2]. Publishing the design of a study before the results are available provides an opportunity to reflect critically on the design of the study, irrespective of the results. Also, a design article provides detailed information about the intervention within the trial to care givers.

The lumbosacral radicular syndrome (LRS) is a complex of symptoms related to the lumbosacral nerve roots. The LRS is a disorder with radiating pain in the leg below the knee in one or more lumbar or sacral dermatomes, and can be accompanied by phenomena associated with nerve root tension or neurological deficits (i.e. sensory deficits in the leg, decreased muscle strength in the leg, decreased reflexes, urinary problems) [4-7]. A prolapsed disc is a frequent cause of LRS, but other causes include spinal or lateral recess stenosis, tumours and radiculitis [4,5,7,8]. The incidence of LRS in the Netherlands is estimated at 5 per 1000 persons a year [8].

Most patients seeking medical care in the Netherlands will first visit a general practitioner (GP), who is regarded as the 'gatekeeper' of the health care system. The majority of health problems presented to GPs are treated by the GPs themselves and they are responsible for most referrals to (para) medical specialists. In 1996 the Dutch College of General Practitioners published their clinical guideline for LRS [5]. There is consensus that treatment of LRS in the first six to eight weeks should be conservative. The exact content of the conservative treatment is yet not clear [9]. Since the study of Vroomen et al. [10] bed rest is not regarded a treatment option for LRS anymore.

Primarily, treatment consists of adequate pain medication, giving information about the natural course of LRS, which in general is favourable, and stimulating to continue the normal daily activities of the patient. GPs in the Netherlands largely comply with the recommendations stated in the clinical guideline regarding management in patients with LRS [11]. However, they deviated regarding the referral to physical therapy (PT), almost half of patients with LRS were referred, whereas this was not recommended in the guideline. No specific patients characteristics could be found for the prescription of physical therapy. So, in general practice referral to PT in patients with LRS is common. However, there is a lack of knowledge of the effectiveness of PT in LRS. Therefore, the aim of this article is to present the design of a randomised clinical trial of conservative treatment (general practitioners and physical therapy) in patients with acute LRS.

## Methods/Design

### Aim

**Figure 1:**
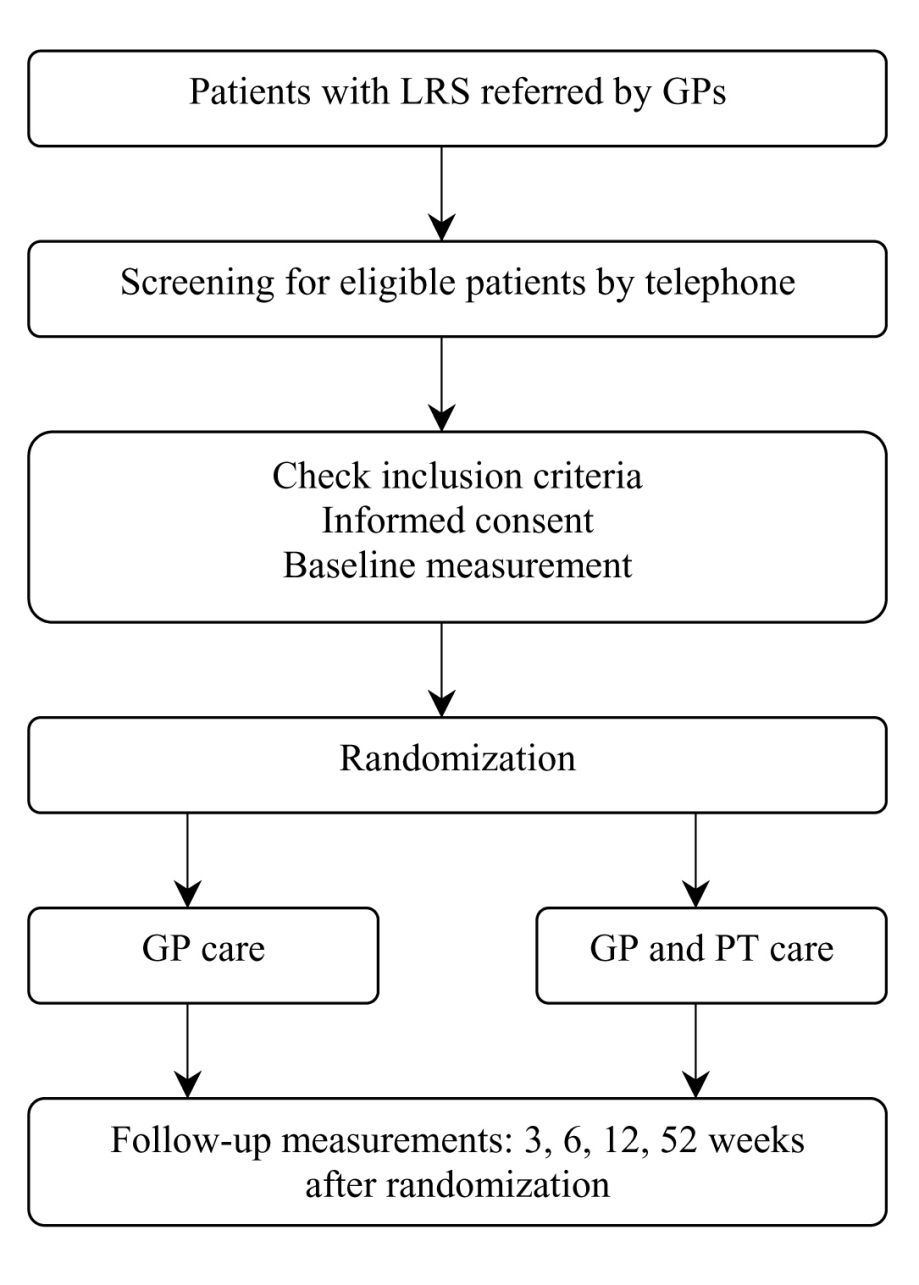
Flow chart of the LRS trial.

The LRS trial aims to assess the effectiveness of PTs management added to GPs management compared to GPs management only in patients with acute LRS. We will use a multicentre, randomised clinical trial as study design. Figure 1 shows the flow chart of the proposed design of the LRS trial. The procedures and design of this study are approved by the Erasmus Medical Center Ethics Committee.

### Study population

**Figure 2:**
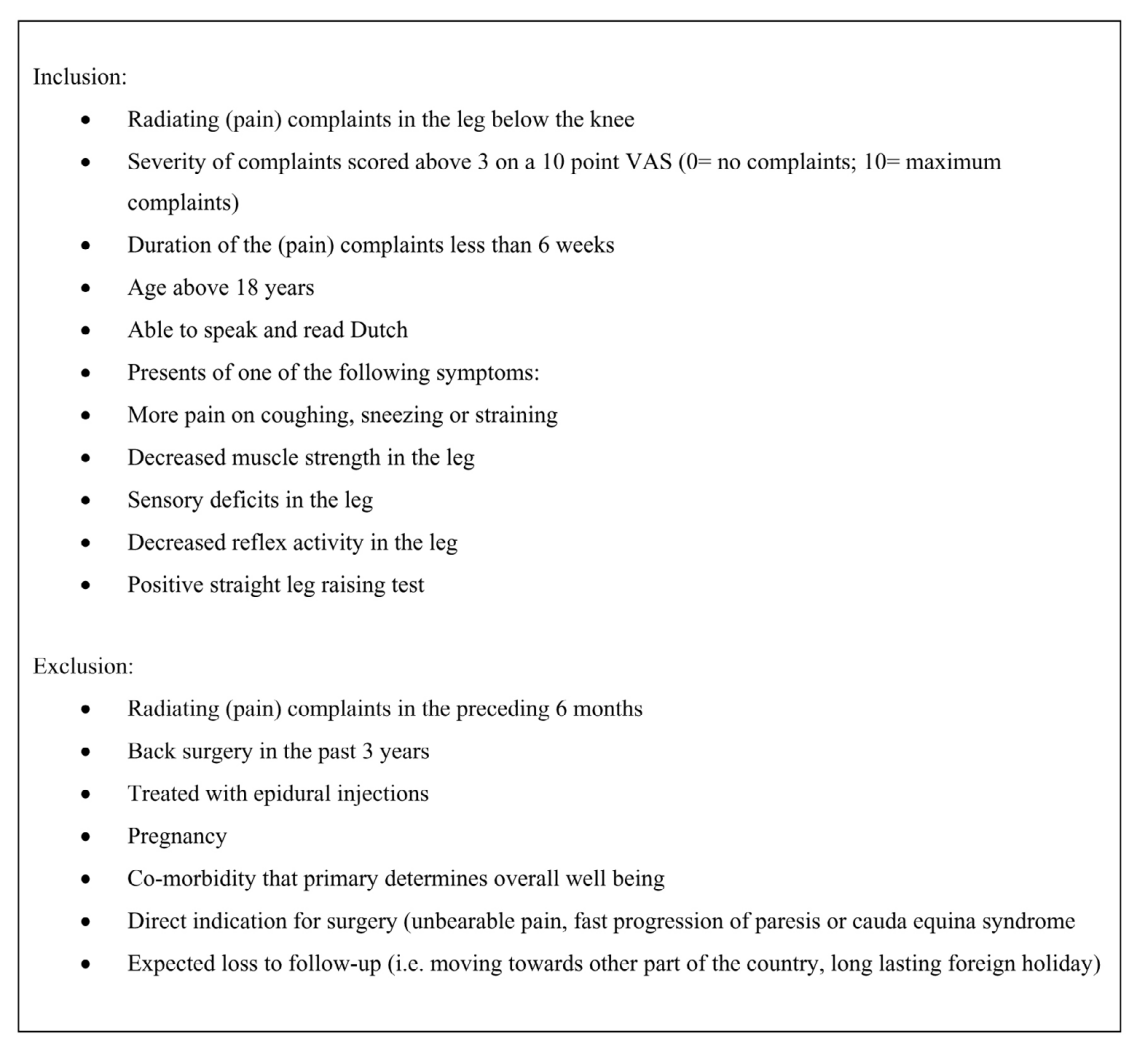
Selection criteria for trial eligibility.

Participating GPs in and around Rotterdam, the Netherlands, will invite patients with suspected acute LRS to participate in the trial. GPs will invite patients from May 2003 till November 2004 if they have radiating (pain) complaints in the leg below the knee; duration of the (pain)complaints is less than 6 weeks, the age is above 18 years and they present one of the following symptoms: more pain on coughing, sneezing or straining, decreased muscle strength in the leg, sensory deficits in the leg, decreased reflex activity in the leg or a positive straight leg raising test. Patients will receive a letter of information about the LRS trial from their GP. Patients' name and telephone number will be faxed to the research institute. Subsequently, a researcher (PL) will screen eligible patients by telephone and make an appointment to check inclusion and exclusion criteria, to complete the informed consent procedure and to perform the baseline measurement. Figure 2 shows the criteria that must be fulfilled to participate in the LRS trial. A research assistant will check these criteria during patients first visit. The informed consent procedure is completed when patients meet the criteria, are willing to participate and give their written consent. Hereafter, the baseline measurement will take place.

### Randomisation

Randomisation will take place after baseline measurement by the research assistant. We use a concealed randomisation procedure using a computer generated randomisation list developed by an independent person. Patients' specific and unique trial number will be typed in a special developed database (i.e. not editable for research assistant and a second randomisation action using the same trial number is not possible) and the random allocation will appear on screen. In order to prevent unequal treatment group sizes, block randomisation will be used with blocks of 10 patients [12]. This means that after every 10th patient the number of patients allocated to both treatment groups is equal. Towards every randomised patient will be explained that the management of their complaint by his or her GP will be continued. Patients who are allocated to physical therapy will be shown a list of participating physical therapists of which he or she can make a choice. The research assistant makes the first appointment with the physical therapist most easily accessible by the patient.

### Blinding

For obvious reasons GPs and PTs are not blinded for treatment allocation. But they are not involved with treatment effect measurements. The patients cannot be blinded because of the ethical reasons as stated by the Medical Ethical Committee. The researcher is involved in the statistical analysis, but the analysis and interpretation of the findings will be audited and verified by an independent and not involved statistician. In this trial the primary outcome measurement and most of the secondary outcome measurements will be scored by the patients. Studies from Ostelo et al [13] and Scholten-Peeters et al [14] mentioned that in this type of study patients are blinded to a certain extent because they are unaware of the exact content of both treatments or may be called naive to the content of the treatment not received. Other more or less similar designed trials from Vroomen et al [10] and Hofstee et al [15] reported that it is not possible to blind participating patients for allocated treatment. Therefore, we think it is important to know any treatment preference of the patients at baseline. Supplementary analysis may show to what extent this effects the scores on outcome measurements of the patients.

### GP intervention

**Figure 3:**
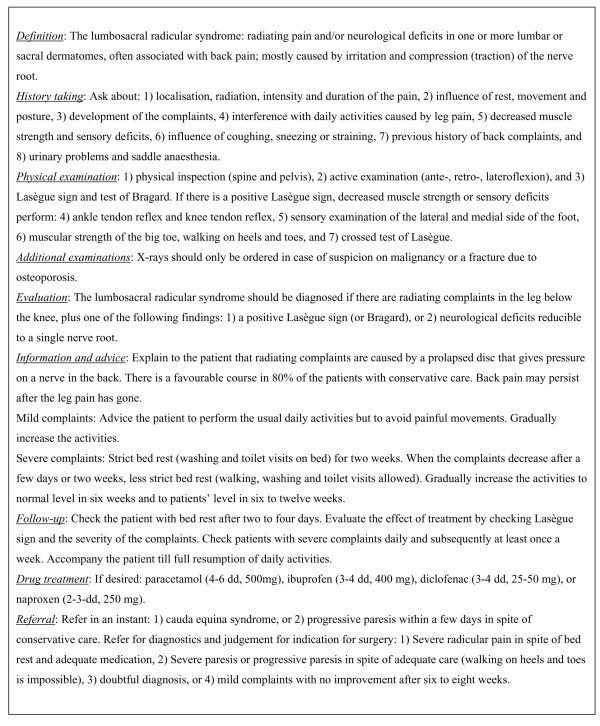
Summary of the clinical guideline 'Lumbosacral radicular syndrome' of the Dutch College of General Practitioners (1996).

All patients will be treated by the GP according to their clinical guideline (see Figure 3). GPs will give information and advice about LRS. If necessary they prescribe adequate pain medication. We asked the GPs not to refer patients to paramedical specialists (i.e. manual therapist, physical therapist, exercise therapist, etc.). Referral to PT is based on randomisation and performed by the research assistant.

### PT intervention

PT treatment will imply information and advice about LRS and exercise therapy. Passive modalities such as massage, manipulation techniques or applying applications (e.g. ultra sound or current waves) are not allowed in the PT treatment. This PT treatment protocol was accomplished in a consensus meeting with participating PTs. The PT will report what kind of information/ advice and what type of exercise the patient receives in each session. Both GP and PT intervention will be restricted to a maximum of 9 treatments/ consultations in the first 6 weeks after randomisation.

### Theoretical background

In the Netherlands, PTs are mainly taught the biomechanic model [13]. This model focuses on somatic issues; it assumes a causal relation between tissue damage and pain. PT could be of additional value in the management of patients with LRS because PTs are 'the experts' in treating musculoskeletal disorders with exercises and advice/ information. The pain reported by a patient is used as guidance to determine the intensity of the exercises and the advice about resuming normal daily activities and work. This study assumes that focussing on (pain) complaints with exercises and advice is the optimal PT treatment in the acute phase (0 to 6 weeks) of LRS.

It is possible that patients may suffer from a fear of movement because of pain [16]. Good advice/ information will reassure these patients and exercises will show them that movement is possible. So, the secondary treatment goal of the PT is to decrease the possibly present fear of movement in these patients.

### Sample size

This trial attempts to enrol 182 patients with LRS, 91 patients in both treatment groups. This sample size is regarded sufficient to detect a difference of 20% (with a α of 0.05 and a power of 80%) in the primary outcome (GPE) between the two treatment groups. A difference of 20% is considered to be clinically relevant [17].

### Measurements

**Figure 4:**
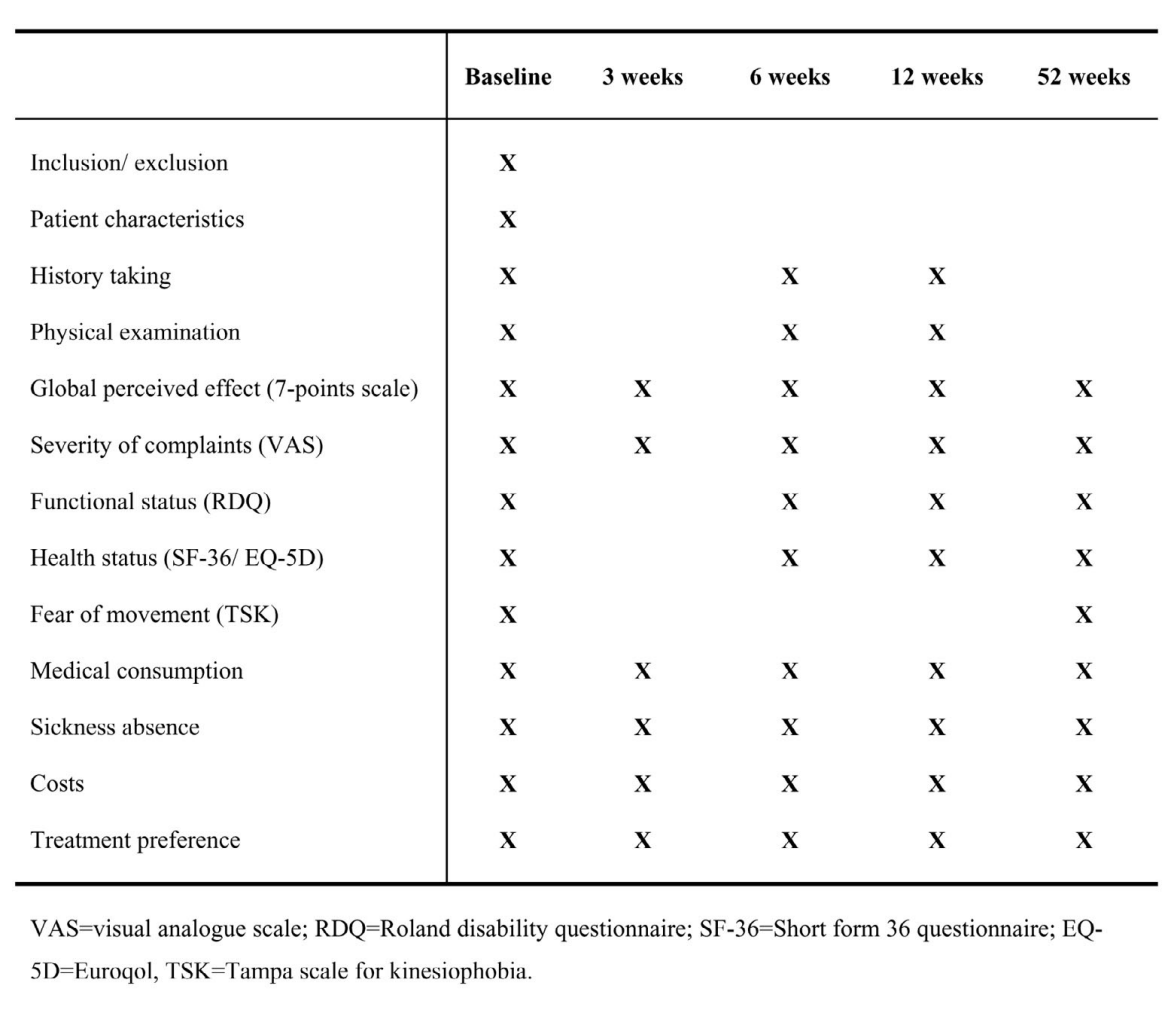
Data collection and outcome measures.

Figure 4 shows the outcome measures and the points of time they are collected. At baseline we will collect patients characteristics such as gender, date of birth, height and body weight. In standardised history taking there will be established whether patients are familiar with LRS in the past, report more pain in the leg on coughing/ sneezing or straining, on sitting, standing, walking and lying down, and if patients report a decreased muscle strength and sensory deficits in the leg. The physical examination consists of the straight leg raising test, the crossed straight leg raising test, test of Bragard, finger-floor distance, standing on toes and heels, knee tendon reflex, ankle tendon reflex, strength of m. extensor hallicus longus, sensory tests (touch, sharp and blunt) in the dermatomes L5/ S1 in the feet.

### Primary outcome measure

The primary outcome measure is the Global Perceived Effect (GPE), measured on a 7 points scale ranging from 1 = completely recovered to 7 = vastly worsened. It is regarded a clinical relevant outcome measure and is regarded valid and responsive to measure the patients' perceived benefit [18-20].

### Secondary outcome measures

Pain severity of the leg and the back will be scored on a 11 points Visual Analogue Scale (VAS) ranging from 0 = no pain to 10 = unbearable pain. Reliability, validity and responsiveness of the VAS have been shown [21-23].

The functional status will be measured with the Roland Morris Disability Questionnaire (RDQ) for sciatica [24]. The scoring of the RDQ is achieved by counting the number of positive responses: a patient individual score can vary from 0 (no disability) to 24 (severe disability). The RDQ has proved to be a valid instrument and appears to be responsive for clinical relevant changes [20,25-28].

Health status will be measured by the 36-item short form (SF-36) [29] and the Euroqol (EQ-5D) instrument [30,31]. Validity and responsiveness on both SF-36 [32-34] and EQ-5D [35-37] proved to be sufficient.

Fear of movement will be measured by the Tampa scale for kinesiophobia (TSK) [38,39]. The TSK consists of 17 items; each rated on a 4-point likert scale. The TSK has been shown to be a valid and responsive instrument [40,41].

Costs will be calculated and include LRS related sickness absence from work, medical consumption (i.e. medication use, additional therapies, visits to health care providers), out-of-pocket expenses and paid help. Patients' treatment preference will be evaluated at baseline and at 4 follow-up measurements.

### Statistical analysis

Baseline comparability will be investigated by descriptive statistics to examine whether randomisation was successful. If necessary, adjustments for baseline variables will be performed in the analysis. Group differences and 95% confidence intervals will be calculated for all outcome measures. The statistical analysis will be performed according tot the intention-to-treat principle, analysing the patients in the treatment group to which they were randomly allocated. Between group differences will be calculated using the Student t-test for continuous variables or Chi-Square for dichotomous variables. In addition a per-protocol analysis will be performed, analysing only those patients with no serious protocol deviations. Comparing the results of the intention-to-treat and the per-protocol analysis will indicate if and to what extent protocol deviations might have biased the results. Multivariate regression analysis will be conducted to examine the influence of baseline variables on outcome.

## Discussion

This article introduces a design of a RCT to evaluate the additional effectiveness of PTs management added to GPs management in patients with LRS. The study is designed in a way that GP and PT treatment is transparent (according a guideline and a consensus meeting) and not a complete "black box". The results of this trial will contribute to the decision of the GP regarding referral of patients with LRS to PT. The inclusion of patients will run until the end of the year 2004. The follow-up measurements will be completed in the end of the year 2005.

## Pre-publication history

The pre-publication history for this paper can be accessed here:
